# Supplementary material for: Single-cell mass cytometry and transcriptome profiling reveal the impact of graphene on human immune cells
Source: Nat Commun. 2017 Oct 24;8:1109. doi: 10.1038/s41467-017-01015-3 (PMC5653675; doi:10.1038/s41467-017-01015-3)
Supplement: Supplementary file 1 — Supplementary Information [file 41467_2017_1015_MOESM1_ESM.pdf]

Description of Supplementary Files

File Name: Supplementary Information
Description: Supplementary Figures and Supplementary Table

File Name: Supplementary Data 1
Description: List of genes differently expressed among the classes passing the cut-off value in T cells. The genes are tabulated along with parametric p-value, FDR, the geometric mean of intensities, fold change and links to major annotation sources.

File Name: Supplementary Data 2
Description: List of genes differently expressed among the classes passing the cut-off value in Monocytes. The genes are Pag. 5 di 5 tabulated along with parametric p-value, FDR, the geometric mean of intensities, fold change and links to major annotation sources.

File Name: Supplementary Data 3
Description: List of the different gene sets identified in T cell, performing the gene set comparison tool in BRB Array-Tools as a scoring test to assign the functional category definitions according to the Gene Ontology Database.

File Name: Supplementary Data 4
Description: List of the different gene sets identified in Monocytes, performing the gene set comparison tool in BRB ArrayTools as a scoring test to assign the functional category definitions according to the Gene Ontology Database.

File Name: Peer Review File

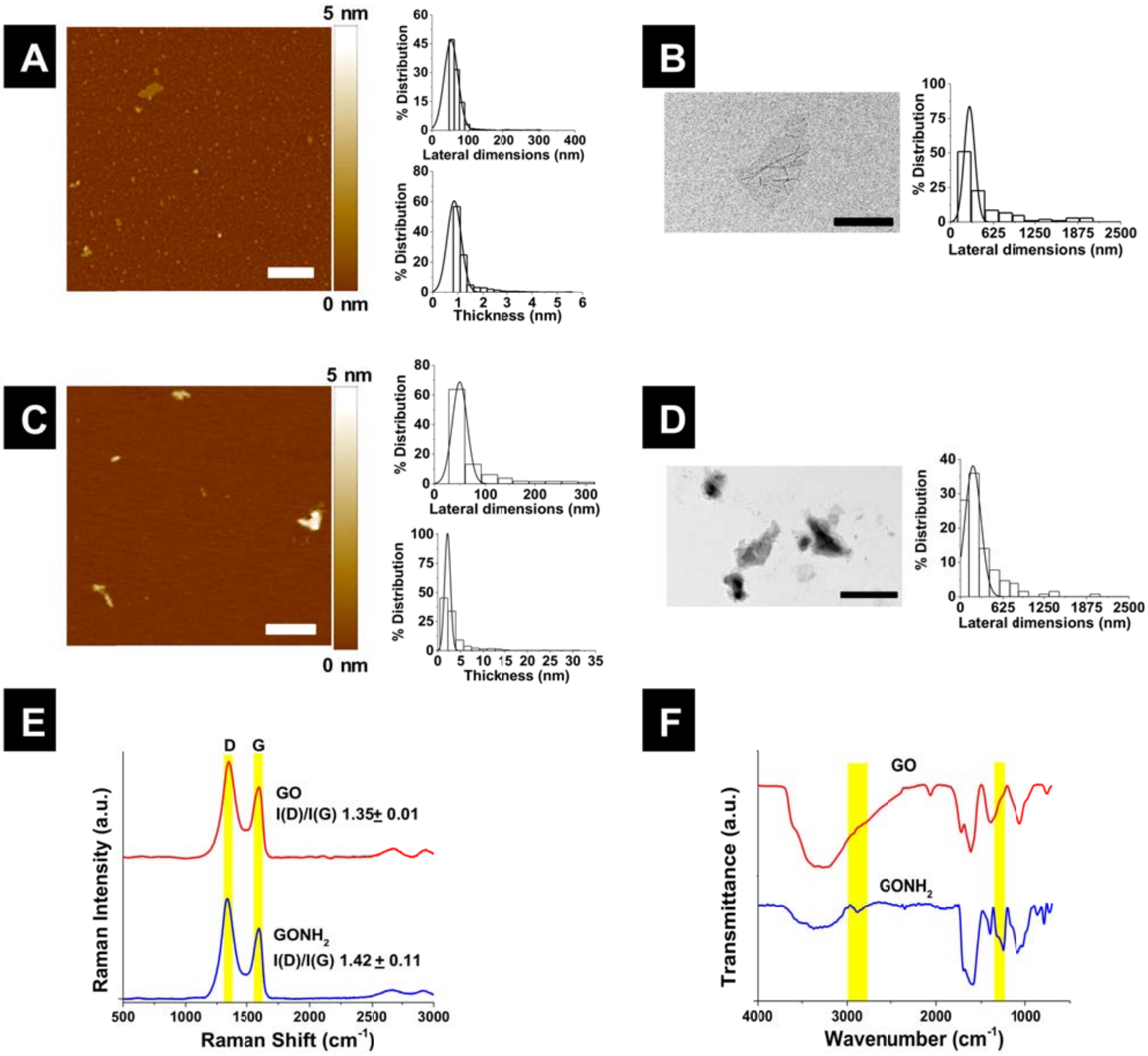

G

Parameter	Technique	GO	GONH$_2$
Lateral dimension	TEM	245.7 ± 57.1 nm (50 nm – 1 µm, n = 106)	184.4 ± 115.4 nm (50 nm – 1 µm, n = 127)
	AFM	52.6 ± 19.1 nm (50 nm – 300 nm, n = 1285)	49.7 ± 14.5 nm (50 nm – 590 nm, n = 748)
Thickness	AFM	0.9 ± 0.3 nm (0.5 nm – 5.5 nm, n = 1285)	2.1 ± 0.6 nm (0.4 nm – 10 nm, n = 748)
I(D)/I(G)	Raman	1.35 ± 0.01	1.42 ± 0.11

Supplementary Figure 1. Physicochemical characterization of GO and GONH$_2$. GO structural characterization using **A**) AFM, followed by lateral dimension and thickness distributions (Scale Barr =500nm); **B**) TEM with lateral dimension distribution (Scale Barr =500nm). GONH$_2$ characterization using **C**) AFM, followed by lateral dimension and thickness distributions (Scale Barr =500nm); **D**) TEM micrograph with lateral dimension distribution (Scale Barr =500nm). (**E**) Raman spectra of GO (red) and GONH$_2$ (blue). F) FT-IR spectra of GO (red) and GONH$_2$ (blue). **G**) Summary of the characterization. Data are presented as mean ± SD. Microscopy data are accompanied by the range and number of particles analyzed.

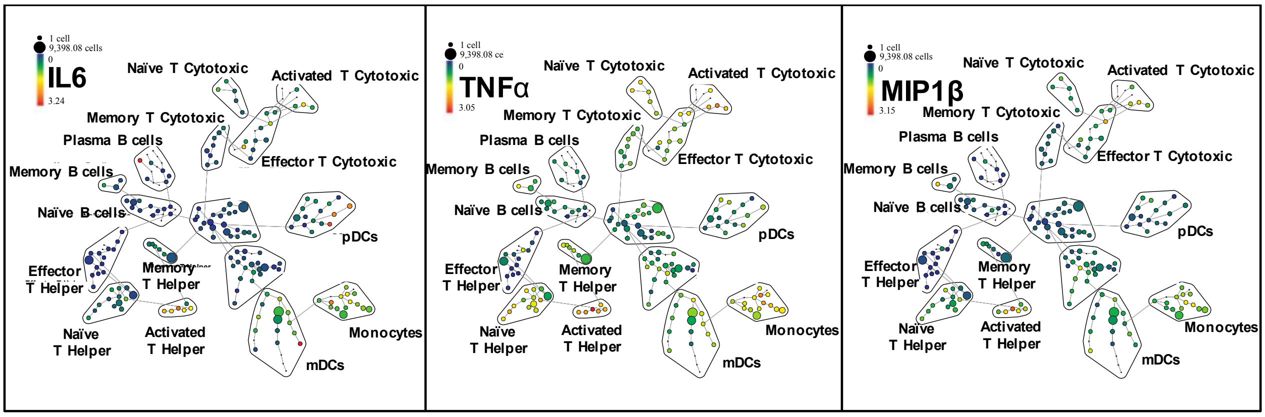

Supplementary Figure 2. Summary of SPADE analysis. SPADE tree plots of untreated samples (CTRL) for significantly expressed cytokines (IL6, TNFα and MIP1β).

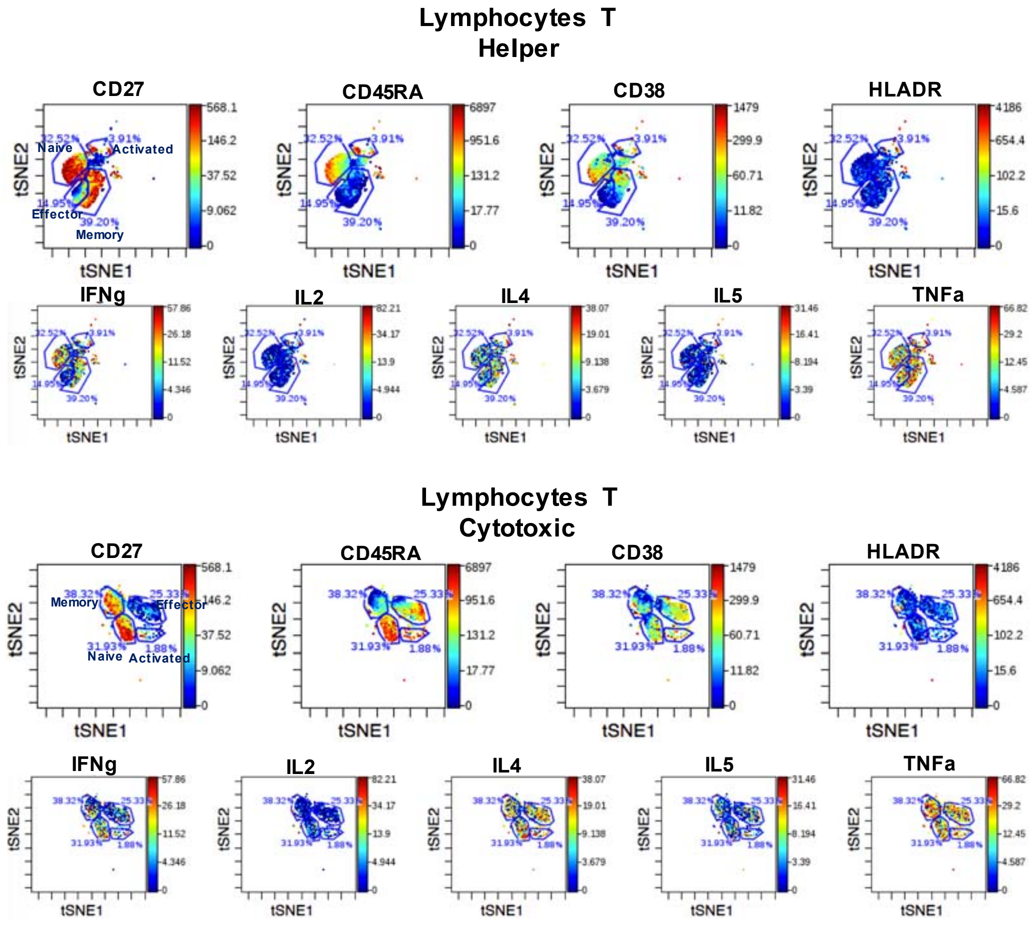

Supplementary Figure 3. Single-cell characterization on gated T lymphocyte subpopulations in GO treated samples (viSNE analysis). Plots show the use of viSNE to obtain a comprehensive single cell view and to distinguish the T cell subpopulations in the GO treated cells. Plots show expression of 9 proteins including cytokines.

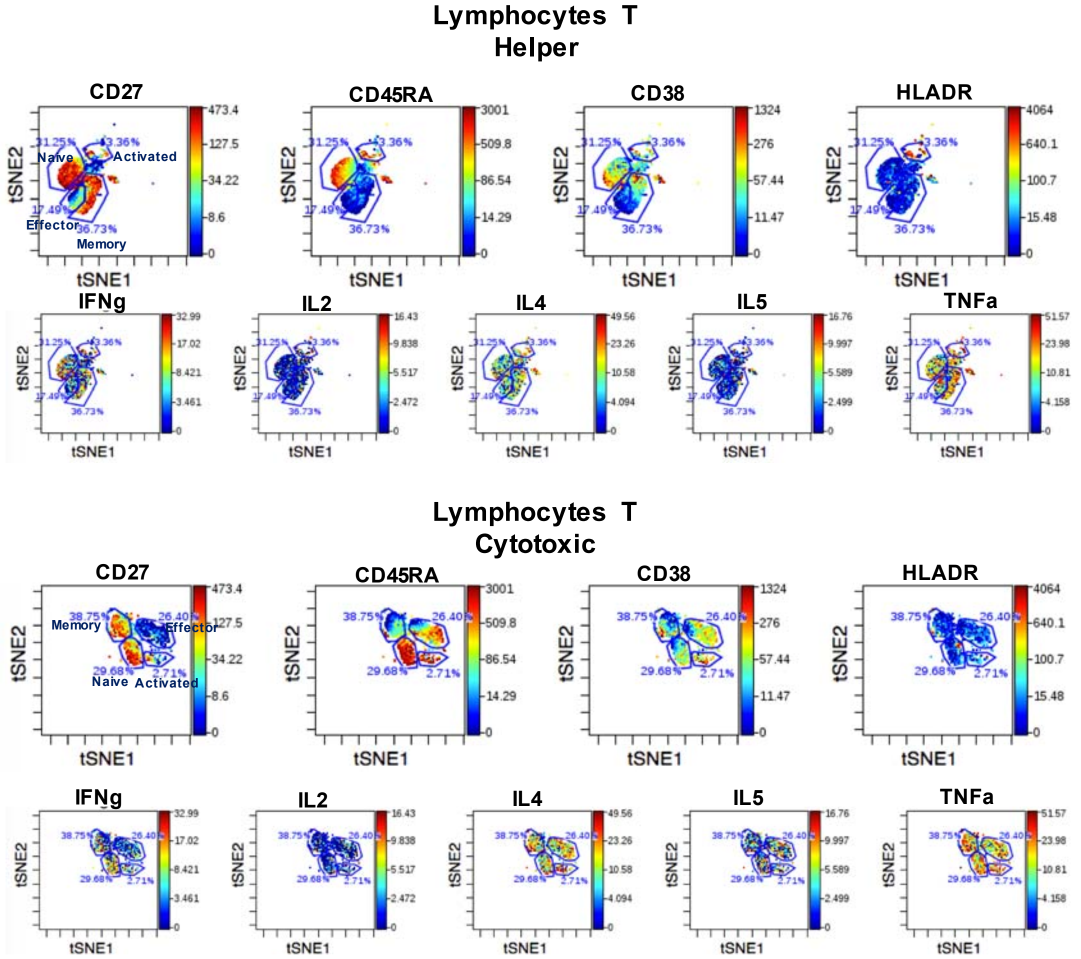

Supplementary Figure 4. Single-cell characterization of gated T lymphocyte subpopulations in $GONH_2$ treated samples (viSNE analysis). Plots show the use of viSNE to obtain a comprehensive single cell view and to distinguish the T cell subpopulations in the $GONH_2$ treated cells. Plots show expression of 9 proteins including cytokines.

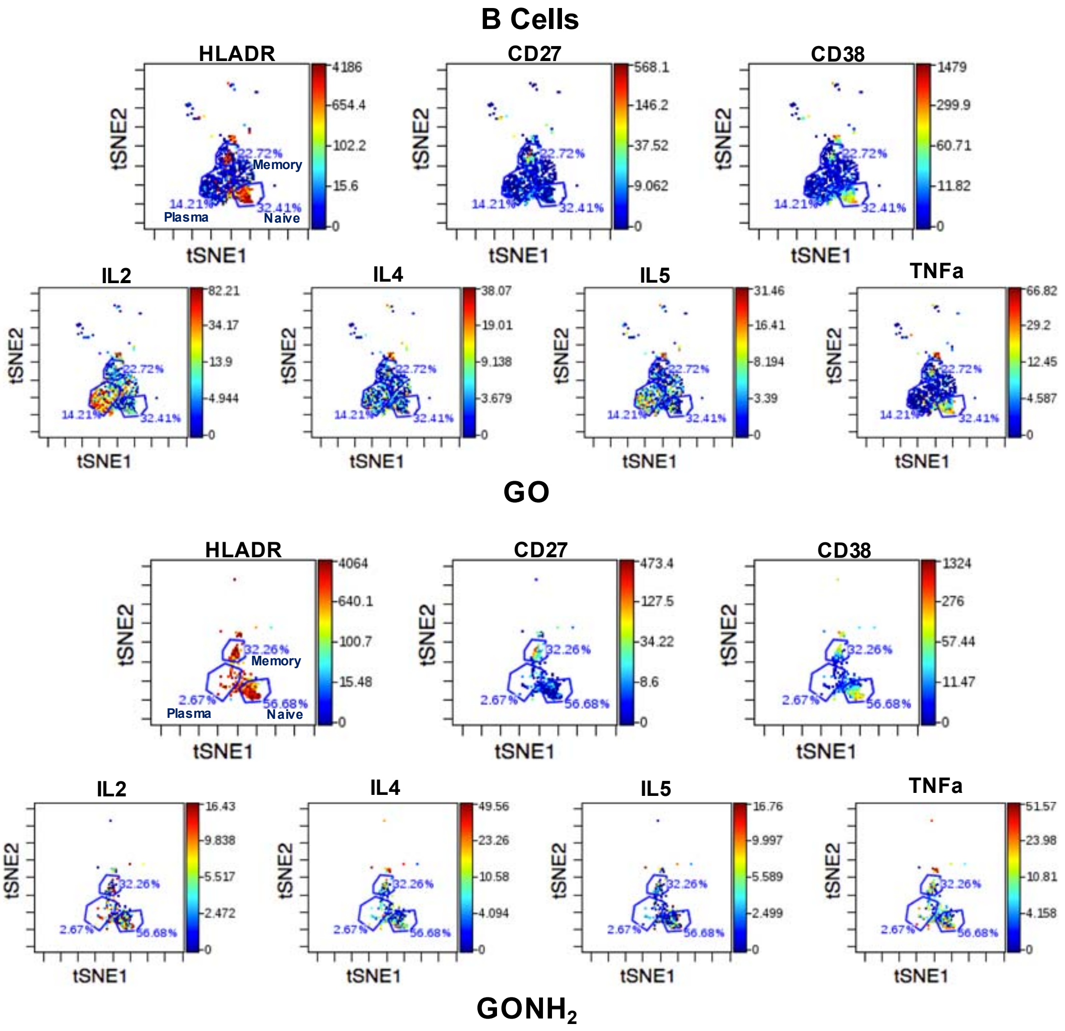

Supplementary Figure 5. Single-cell characterization of GO and $GONH_2$ on gated B cells (viSNE analysis). Plots show the use of viSNE to obtain a comprehensive single-cell view and to distinguish the B cell subpopulations in the GO and $GONH_2$ treated cells. Plots show expression of the 7 proteins used to distinguish the single population including cytokines.

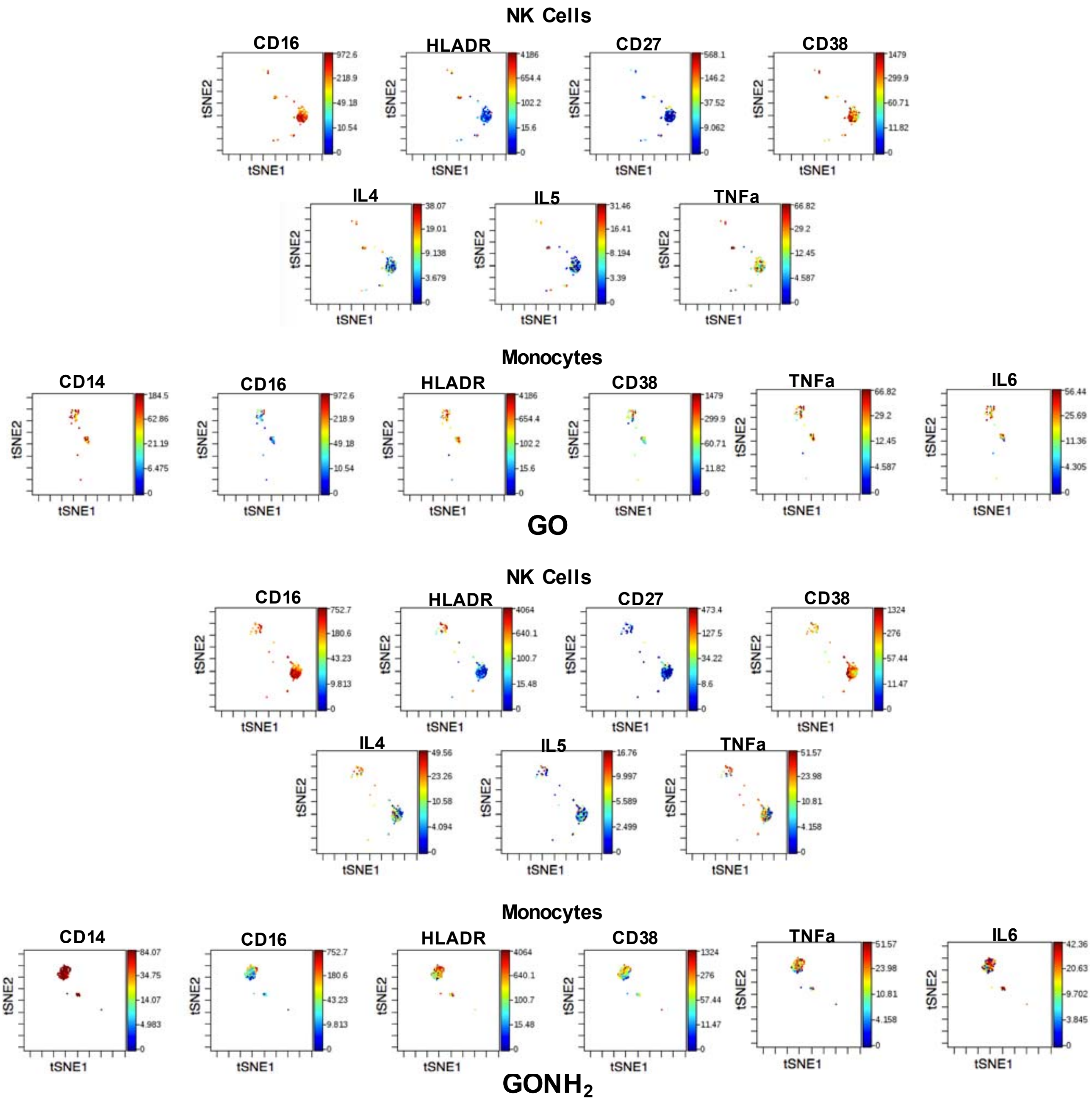

Supplementary Figure 6. Single-cell characterization of gated NK cells and monocytes in GO and $GONH_2$ treated samples (viSNE analysis). Plots show the use of viSNE to obtain a comprehensive single-cell view and to distinguish the NK cells and monocytes in GO and $GONH_2$ treated cells. Plots show expression of 9 proteins including cytokines for NK cells and 6 for monocytes.

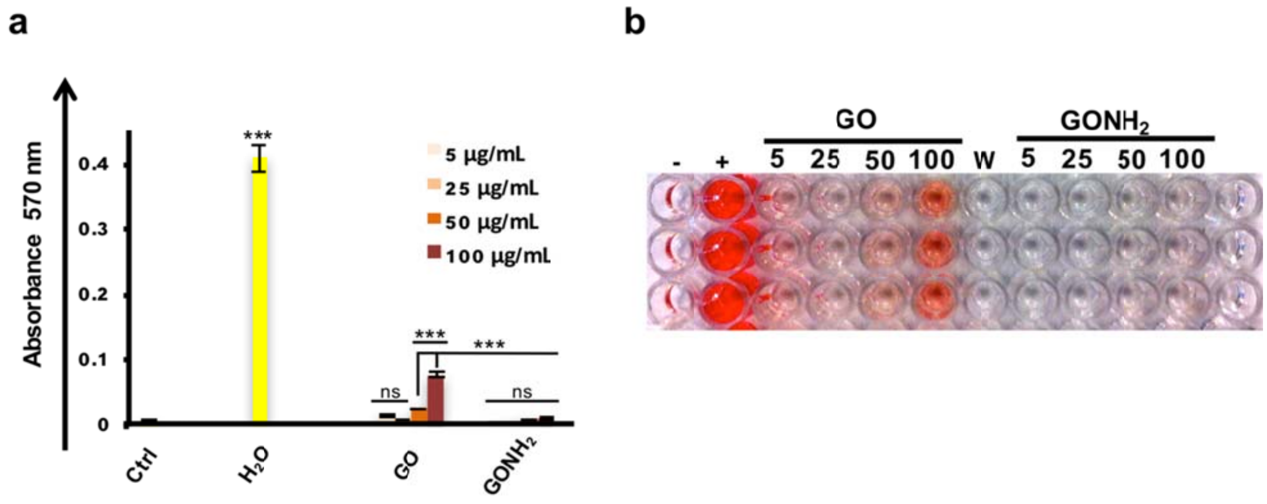

Supplementary Figure 7. Hemolysis analysis. GO and $GONH_2$ were incubated (at increasing concentrations: 5, 25, 50, 100 µg·mL^{-1}) with red blood cells (RBCs) at 25°C for 2 h. **a**) Sample absorbance measured at 570 nm. **b**) Pictures of human RBCs treated with the different GOs. The red color of the solution is due to the release of hemoglobin from the damaged RBCs. PBS mixed with RBCs (-) and ultrapure water mixed with RBCs (+) served as negative and positive control, respectively. PBS alone was used as additional control (W). The experiments were performed in triplicate (***= P value <0.0001, ns= P value >0.05 Statistical analysis performed by two-way ANOVA and Tukey's multiple comparison test. Where not specified the P value is intended vs. Ctrl).

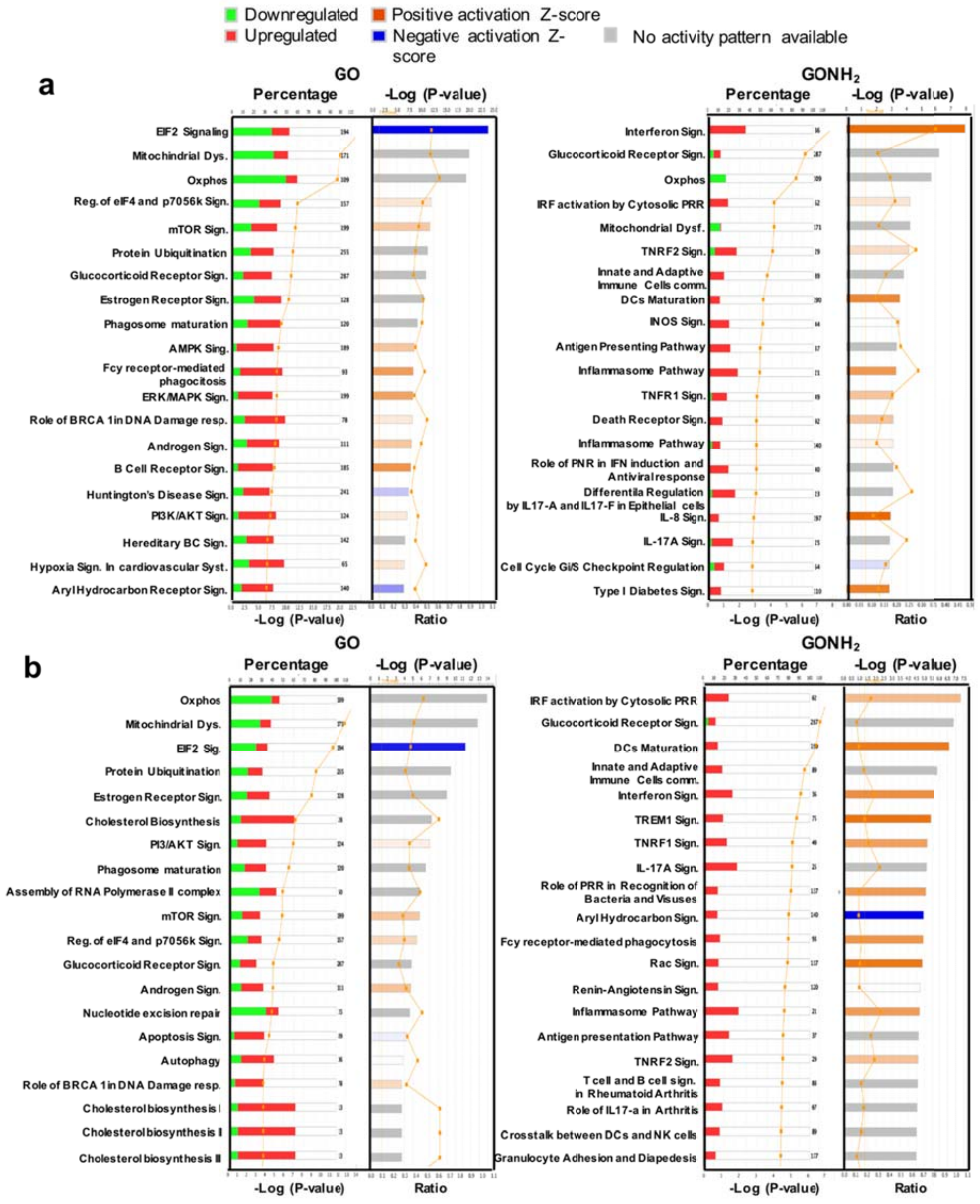

Supplementary Figure 8. Ingenuity pathways analysis. Top 20 canonical pathways and Z-score ranking according to significance level [Fisher exact test -log (p-value)] modulated by GO and $GONH_2$ in **a**) T-cells and **b**) monocytes identified using IPA software.

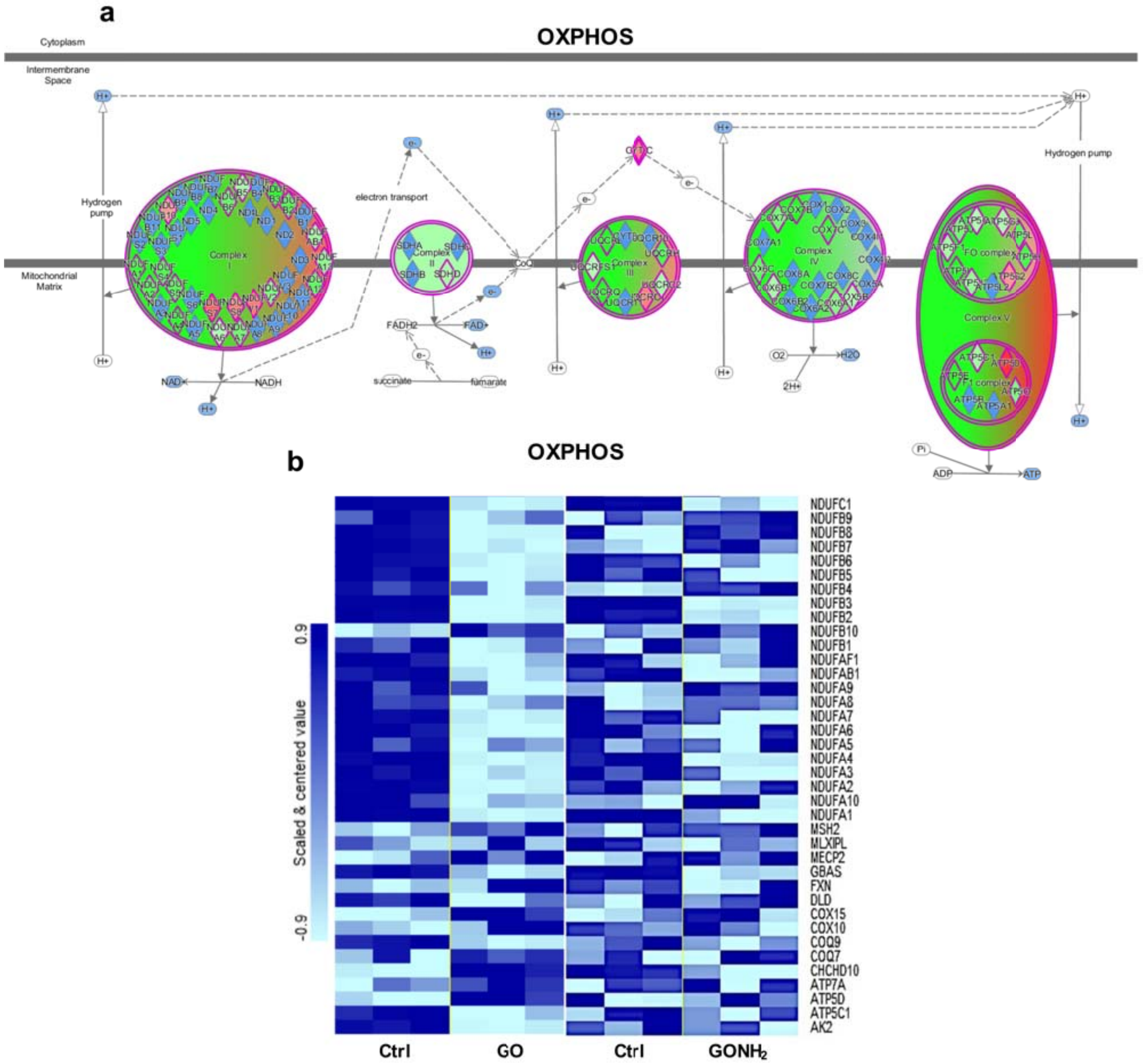

Supplementary Figure 9. Gene set enrichment with Gene Ontology and IPA analysis. a) OXPHOS pathway found modulated in GO sample. **b)** Gene ontology visualizationheatmap of OXPHOS pathway after treatment with GO and GONH2 in T-cells.

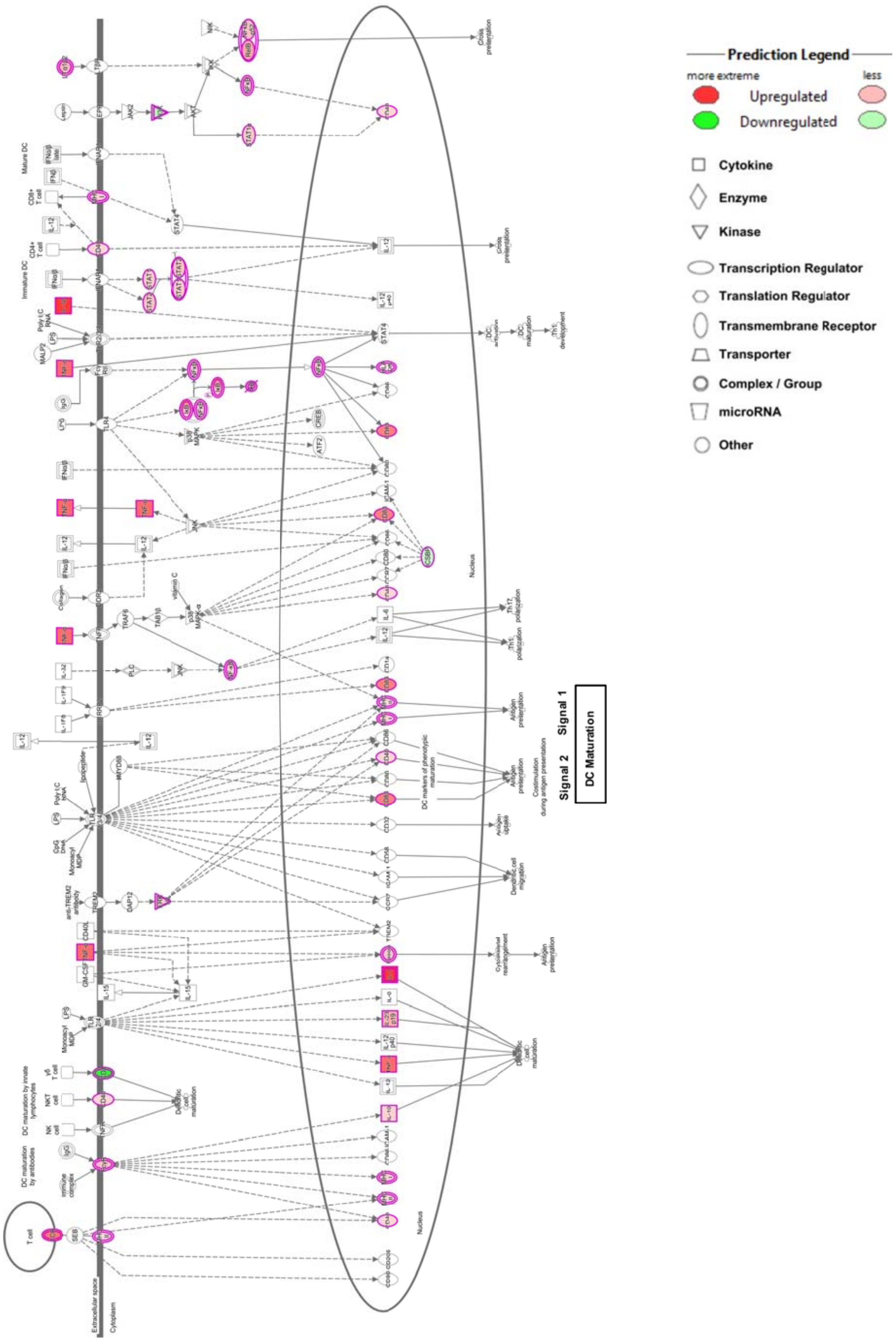

Supplementary Figure 10. Significant canonical pathways. DC maturation pathway in monocytes after treatment with $GONH_2$.

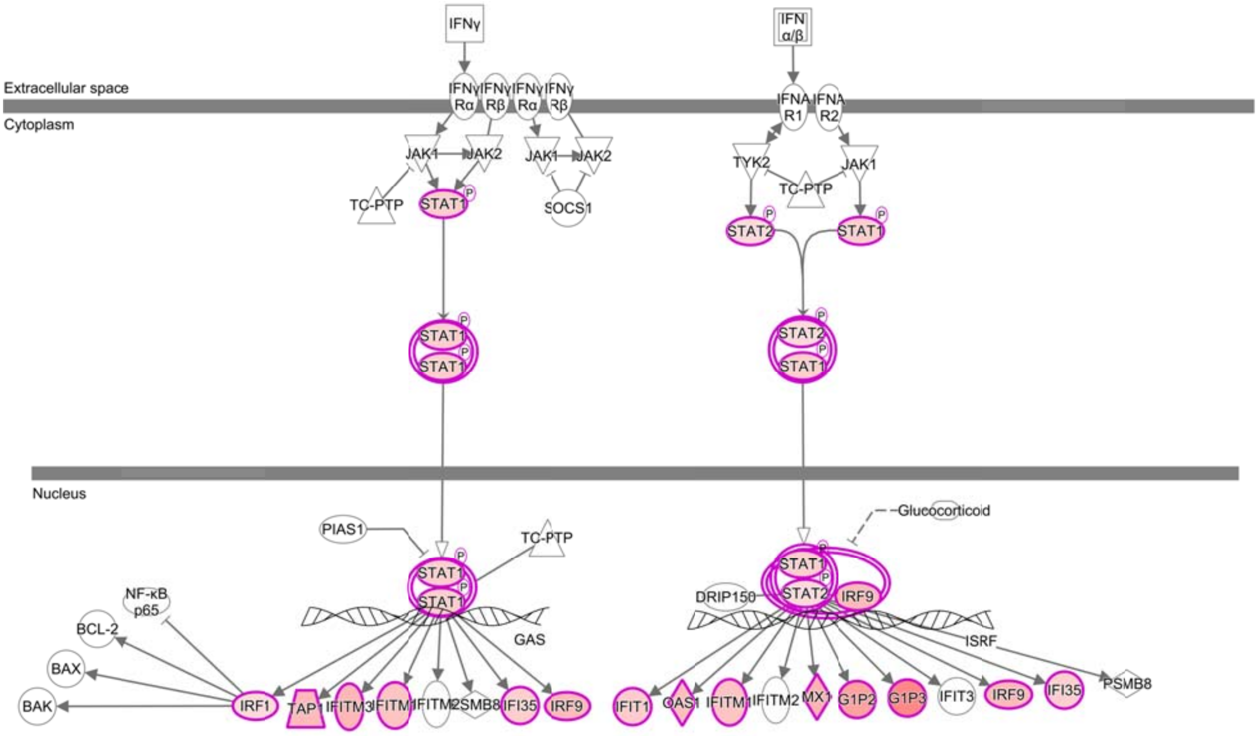

Supplementary Figure 11. Significant canonical pathways. IFN signaling pathway in T-cells after treatment with $GONH_2$ (refer to Supplementary Figure 10 legend).

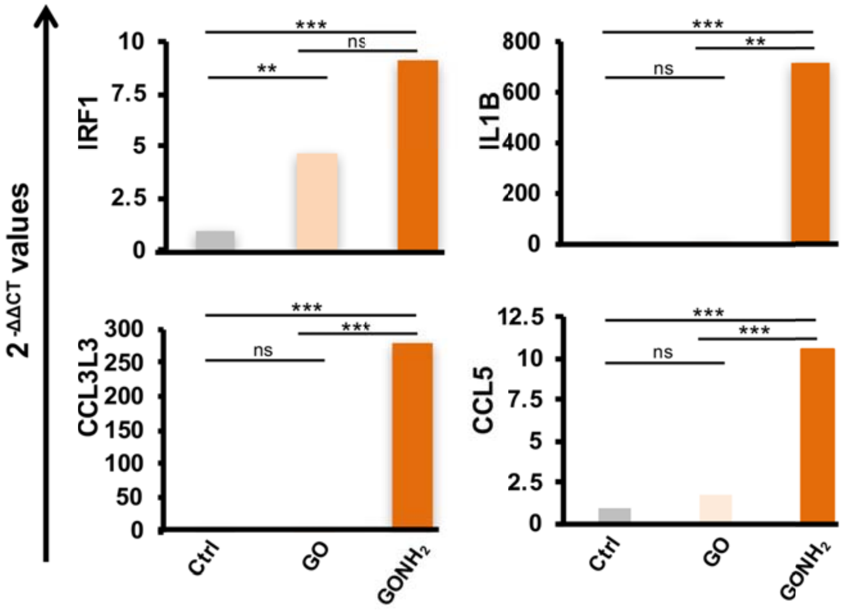

Supplementary Figure 12. Microarray data validation in monocytes cell line. mRNA levels were measured in triplicate by quantitative real-time PCR. Histograms show residual mRNA level expressed as $2^{-\Delta\Delta ct}$ that gives the measure of fold changes between GO and $GONH_2$ and controls (always expressed as 1) (**= P value < 0.001, ***= P value <0.0004 Statistical analysis performed by one-way ANOVA test with Bonferroni correction).

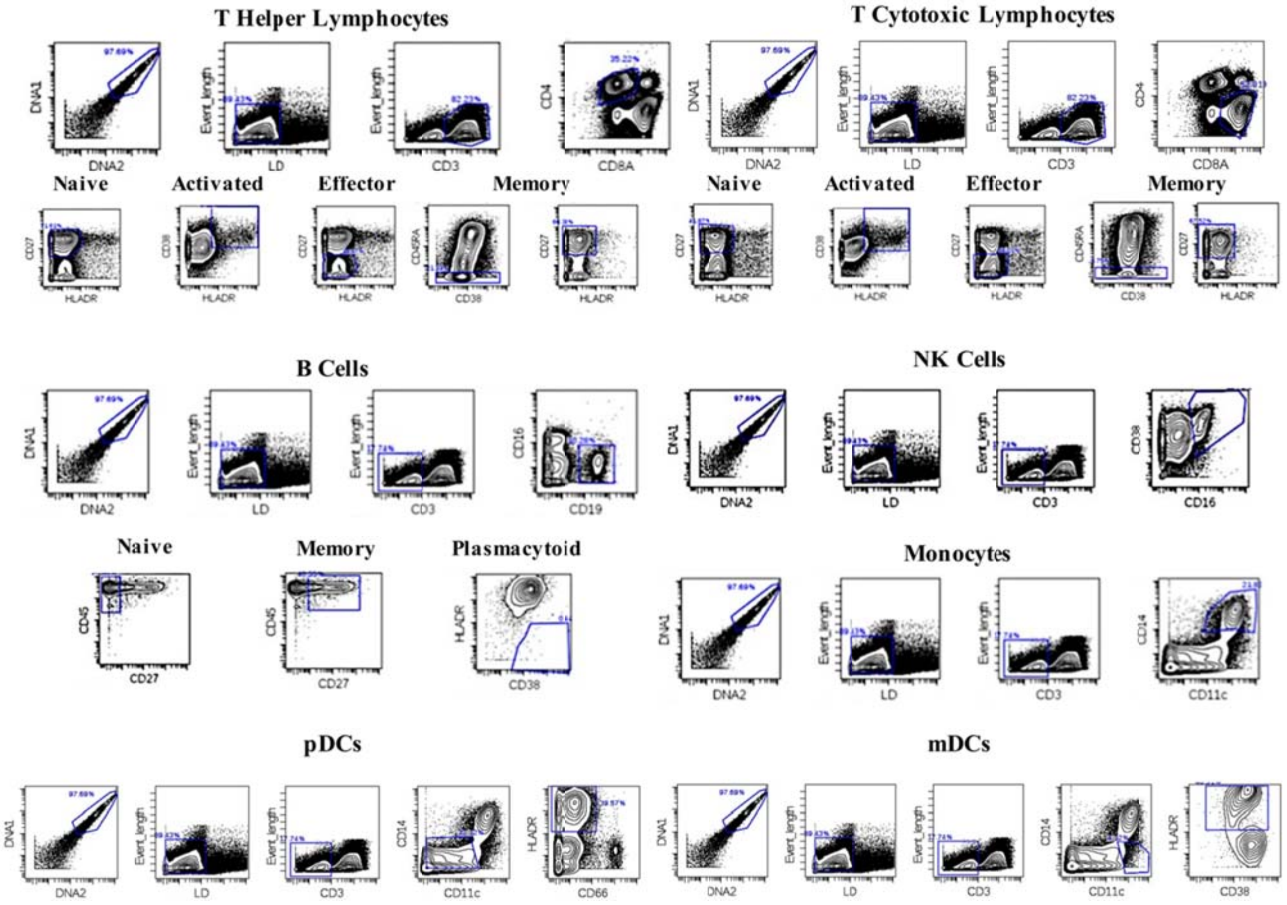

Supplementary Figure 13. Gating strategy of different immune cell subpopulations. The used gating strategy for the identification of the different immune cell subpopulations is shown for the control sample as a contour dot plots. The gating was performed following the Fluidigm instruction of the Maxpar panel used. The cell events discrimination was performed first using Ir 191 Di(DNA1) and Ir 193 Di (DNA2), LD stands for CIS.

	GO	GONH$_2$	Tukey's multiple comparisons test	GO		GONH$_2$		
CTL	Events			Mean Diff	95% CI of diff	Mean Diff	95% CI of diff	Significance for Apha 0.05
Run 1	8574	23846	Run 1 vs.Run 2	132	-23760 to 24024	-5343	-15392 to 4706	ns
Run 2	8442	29189	Run 1 vs. Run 3	31	-23861 to 23923	-3020	-13069 to 7029	ns
Run 3	8543	26866	Run 2 vs. Run 3	-101	-23993 to 23791	2323	-7726 to 12372	ns
Th	Events			Mean Diff	95% CI of diff	Mean Diff	95% CI of diff	Significance for Apha 0.05
Run 1	38559	49255	Run 1 vs.Run 2	21460	-2432 to 45352	3475	-6574 to 13524	ns
Run 2	17099	45780	Run 1 vs. Run 3	18090	-5802 to 41982	2845	-7204 to 12894	ns
Run 3	20469	46410	Run 2 vs. Run 3	-3370	-27262 to 20522	-630	-10679 to 9419	ns
Monocytes	Events			Mean Diff	95% CI of diff	Mean Diff	95% CI of diff	Significance for Apha 0.05
Run 1	139	320	Run 1 vs.Run 2	9	-23883 to 23901	-5	-10054 to 10044	ns
Run 2	130	325	Run 1 vs. Run 3	-54	-23946 to 23838	-6	-10055 to 10043	ns
Run 3	193	326	Run 2 vs. Run 3	-63	-23955 to 23829	-1	-10050 to 10048	ns
B Cells	Events			Mean Diff	95% CI of diff	Mean Diff	95% CI of diff	Significance for Apha 0.05
Run 1	371916	22486	Run 1 vs.Run 2	-13866	-37758 to 10026	-3179	-13228 to 6870	ns
Run 2	385782	25665	Run 1 vs. Run 3	1032	-22860 to 24924	-738	-10787 to 9311	ns
Run 3	370884	23224	Run 2 vs. Run 3	14898	-8994 to 38790	2441	-7608 to 12490	ns
NK Cells	Events			Mean Diff	95% CI of diff	Mean Diff	95% CI of diff	Significance for Apha 0.05
Run 1	6560	17557	Run 1 vs.Run 2	-389	-24281 to 23503	-197	-10246 to 9852	ns
Run 2	6949	17754	Run 1 vs. Run 3	1187	-22705 to 25079	5892	-4157 to 15941	ns
Run 3	5373	11665	Run 2 vs. Run 3	1576	-22316 to 25468	6089	-3960 to 16138	ns
DCs	Events			Mean Diff	95% CI of diff	Mean Diff	95% CI of diff	Significance for Apha 0.05
Run 1	7574	6661	Run 1 vs.Run 2	2434	-21458 to 26326	1530	-8519 to 11579	ns
Run 2	5140	5131	Run 1 vs. Run 3	-733	-24625 to 23159	-4313	-14362 to 5736	ns
Run 3	8307	10974	Run 2 vs. Run 3	-3167	-27059 to 20725	-5843	-15892 to 4206	ns

Supplementary Table 1. SPADE robustness evaluation among three independent runs. SPADE analysis were run three times with GO and GONH$_2$ samples. The number of major cell population events are reported. Statistical analyses confirming the robustness of the SPADE data were performed using a two-way ANOVA and Tukey's multiple comparison test of every algorithm run.
